# Preventing zoonotic and zooanthroponotic disease transmission at wild great ape sites: Recommendations from qualitative research at Bwindi Impenetrable National Park

**DOI:** 10.1371/journal.pone.0299220

**Published:** 2024-03-01

**Authors:** Maya Homsy King, Haven Nahabwe, Benard Ssebide, Laura H. Kwong, Kirsten Gilardi

**Affiliations:** 1 School of Public Health, University of California, Berkeley, Berkeley, California, United States of America; 2 Church of Uganda Bwindi Community Hospital, Kinkizi Diocese, Kanungu, Uganda; 3 Gorilla Doctors, Mountain Gorilla Veterinary Project Incorporated., Kampala, Uganda; 4 School of Veterinary Medicine, University of California, Davis, Davis, California, United States of America; The University of Hong Kong, CHINA

## Abstract

Employees at wild great ape sites are at high risk of transmitting infectious diseases to endangered great apes. Because of the significant amount of time employees spend near great apes, they are a priority population for the prevention and treatment of zoonotic and zooanthroponotic spillover and need adequate preventive and curative healthcare. Qualitative, semi-structured interviews with 46 staff (rangers and porters) at Bwindi Impenetrable National Park, Uganda (BINP) and key informants from five other wild great ape sites around the world were performed. The objectives of the study were to 1) evaluate health-seeking behavior and health resources used by staff in contact with great apes at Bwindi Impenetrable National Park; 2) evaluate existing occupational health programs for employees working with great apes in other parts of the world; and 3) make recommendations for improvement of occupational health at BINP. Results show that BINP employees do not frequently access preventive healthcare measures, nor do they have easy access to diagnostic testing for infectious diseases of spillover concern. Recommendations include assigning a dedicated healthcare provider for great ape site staff, providing free annual physical exams, and stocking rapid malaria tests and deworming medication in first aid kits at each site.

## Introduction

Transmission of infectious pathogens among nonhuman primates, humans and domestic livestock poses a significant threat to human and domestic animal health and the sustainability of wildlife [1]. This potential transmission is a key element of the One Health approach, which recognizes and emphasizes the interdependence of human, animal and environmental health, and postulates that the health of all must be addressed to achieve full ecosystem health [2]. The risk of human infectious disease crossing over to animals (zooanthroponotic transmission), and animal infectious disease being transmitted to humans (zoonotic transmission) is particularly high for great apes, because of the genetic similarity between humans and great apes [3].

Gorillas and other great ape species are susceptible to human pathogens, including respiratory infections such as measles, human respiratory syncytial virus and human metapneumovirus, which often cause high morbidity and mortality [4–6]. Parasitic infections can also be shared between humans and nonhuman primates including *Plasmodium* spp., *Mycobacterium tuberculosis*, *Giardia duodenalis* and *Cryptosporidium* spp. [7]. Human-origin scabies has been documented in mountain gorillas [8].

Disease outbreaks can be devastating to endangered species. One study that used data from sixteen previous studies on outbreaks among great apes in East and West Africa predicted that recovery time for a gorilla population (time taken for a population to reach initial size again) for a low-mortality (4%) infectious disease outbreak was 5 years. That of a high-mortality infectious disease outbreak, such as Ebola (96% mortality), would be 131 years [9]. The risk of infectious disease to gorilla populations is increasing as habituation to tourism and increasing human populations around their habitats rise. The risk of great apes transmitting pathogens to human populations is also present. The most pertinent example is human immunodeficiency virus, which is thought to have originated from an interaction between a human and a wild chimpanzee in Cameroon during the early 20^th^ century [10].

Mountain gorillas (*Gorilla beringei beringei)*, an endangered nonhuman primate species, are threatened by habitat loss, military and rebel activity, and human pathogens. The last remaining populations survive in Virunga National Park in the Democratic Republic of the Congo, Volcanoes National Park in Rwanda, and Mgahinga Gorilla National Park and Bwindi Impenetrable National Park (BINP) in Uganda [11]. BINP is a popular tourist destination for mountain gorilla viewing and tracking. The park attracted 36,341 tourists in 2019 [12] and it currently employs ~600 staff including rangers, porters and security personnel. ‘Rangers’ in this paper will be used to refer to full-time staff employed by the Uganda Wildlife Authority (UWA) who work in the forest. UWA is the branch of government responsible for the management of national parks. The term encompasses staff who specialize as guides, security personnel and trackers, as well as those who do not. It does not include porters, as they are not formally employed by UWA. The areas around BINP are some of the most densely populated in rural Africa, with over 350 people per square kilometer [13]. Park rangers and porters frequently interact with gorillas through guiding, tracking, and monitoring activities. They also interact daily with each other, tourists, and local communities. These contacts pose a risk of disease transmission between humans and gorillas. A 2003 survey of communities around the park found a high prevalence of infectious disease symptoms, including 72% of people with coughs and 56% with fevers [14]. Early diagnosis and treatment of infectious diseases in humans can lower the risk of zooanthroponotic transmission, and preventative healthcare can maintain good health and prevent zooanthroponotic diseases.

To date, there have been few studies of employee health programs or employee access to healthcare in wild great ape settings. A 2004 study describes a health program in Rwanda for employees working with mountain gorillas, including screenings for tuberculosis (TB), human immunodeficiency virus (HIV) and screening of blood, urine and stool [15]. The program offered yearly retesting and screenings as well as interventions including deworming. The International Union for the Conservation of Nature (IUCN) has published guidelines on great ape health [16] that detail that a standard employee health program should include an annual physical exam administered by a physician, access to health tests including temperature, diagnostic testing, access to vaccines and vaccine verification, deworming medication administered quarterly, referrals if employees have emergency or complicated conditions, and lastly to be provided with relevant health education. The IUCN employee health guidelines are not context-specific, and they are the ideal, assuming that every site has the necessary resources. However most wild great ape sites are located in resource-poor, remote settings, and it is simply not possible to implement employee health programs that reach the standards in the guidelines [17].

This aims of this study were to 1) evaluate health-seeking behavior and health resources used by national park rangers in contact with great apes at Bwindi Impenetrable National Park through qualitative semi-structured interviews; 2) evaluate existing occupational health programs for employees working with great apes in other parts of the world against IUCN employee health guidelines using key informant (KI) interviews; and 3) make recommendations for improvement of occupational health at BINP.

## Methods

### Study design

The study used a cross-sectional qualitative design, as the objective was to gain an understanding of the status of employee health programs at BINP and five other great ape sites. A qualitative design enables a richer description of complex issues, such as individual health-seeking behavior, and allows participants to speak freely on their experiences and recommendations for the healthcare available to them. The study had two parts, detailed below: healthcare access and use for Bwindi Impenetrable National Park staff, and key informant interviews regarding employee health programs at other great ape sites.

### Ethics statement

Institutional Review Board approval was obtained from The AIDS Support Organization (TASO) in Uganda (Ref # TASOREC/105/2022-UG-REC-009), from University of California Berkeley’s Office for the Protection of Human Subjects (Protocol #2022-02-15057) and from the Uganda National Council for Science and Technology (Ref #HS2136ES). Additional approval was obtained from Bwindi Community Hospital (no reference number), and from the Uganda Wildlife Authority (no reference number) to interview their staff. Written informed consent was obtained from all participants. Participants at BINP were given one hard copy of the informed consent form, and the investigators kept a second copy, while KIs signed the informed consent via email.

### Data collection

Gorilla Doctors, a partnership between the UC Davis Wildlife Health Center and the Mountain Gorilla Veterinary Project (MGVP, Inc.), provides veterinary care for the gorillas at BINP and in other mountain gorilla parks, and has been at BINP since 2009. Recruitment of subjects at BINP took place from the June 17 to July 2, 2022. Based on the researcher’s request to interview a certain number of individuals who performed a variety of jobs, Gorilla Doctors conducted purposive sampling and suggested individual rangers at the Buhoma, Ruhija and Nkuringo ranger stations to be interviewed and to recruit other participants for interviews. The rangers at each sector identified by Gorilla Doctors then explained the purpose of the research to their staff and suggested off-duty staff to participate in interviews. A condition of conducting research with UWA staff was that they would recommend potential participants and thus make sure those participating were not on duty or on leave. Rushaga, the fourth sector, was not included due to time and budget constraints. The omission of this sector may have left out important findings in terms of healthcare accessibility and health seeking behavior. On the other hand, Rushaga is not far from Nkuringo, meaning it is unlikely that staff would have had drastically different healthcare facilities accessible to them.

**Fig 1:**
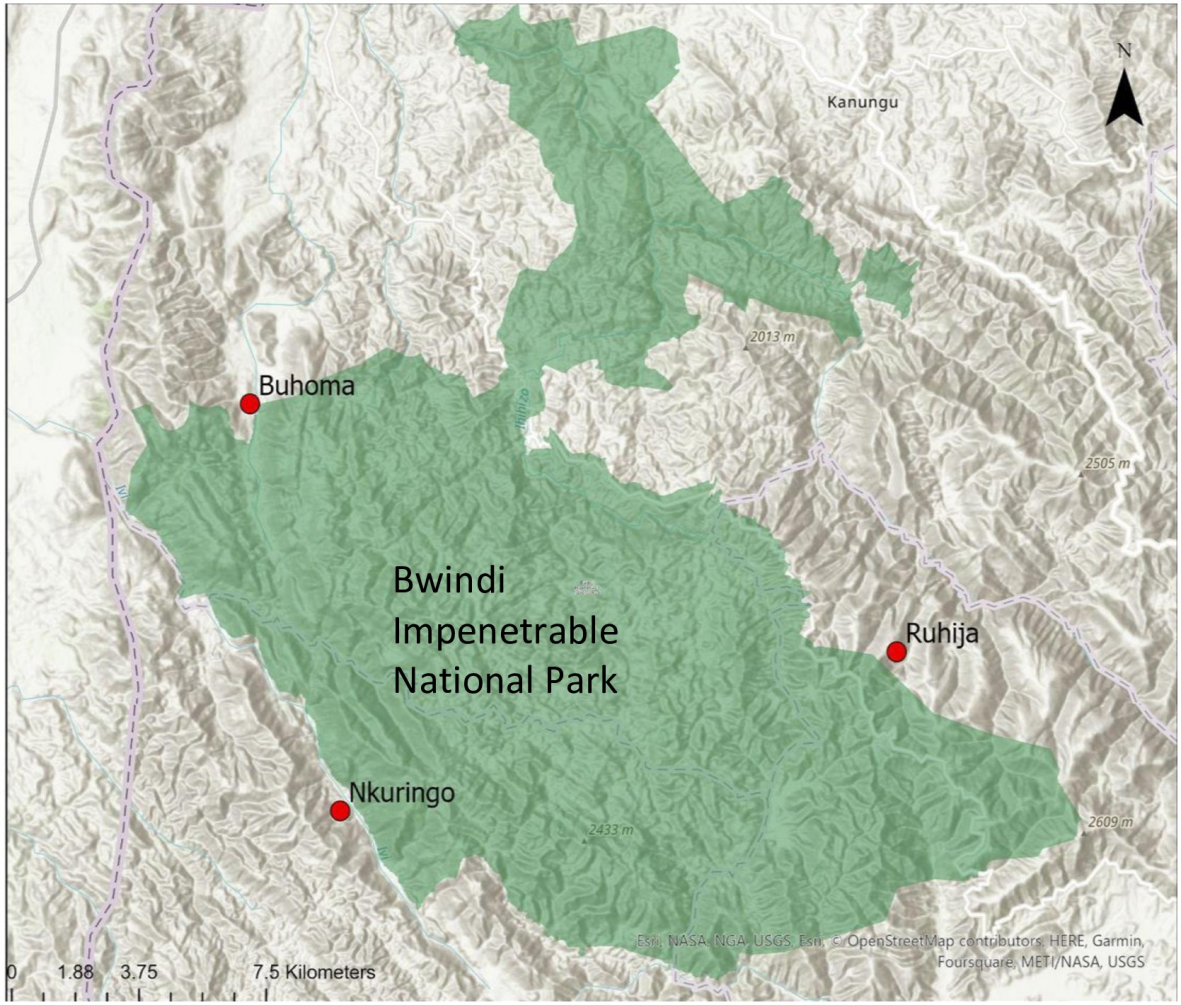
Data collection sites at BINP. Red dots indicate sectors of the park where ranger stations are located, which is where data collection took place. Not pictured is Rushaga station, south of Nkuringo, as data collection did not take place there. © OpenStreetMap contributors.

The sampling aimed to recruit an equal distribution of participants across occupational categories, gender, and stationed sector of the park. The sample size was limited by budget and time constraints and was capped at 50 participants. Buhoma is the sector of the park where the Uganda Wildlife Authority (UWA) headquarters and Bwindi Community Hospital (BCH) are located. Ruhija and Nkuringo sectors are more remote from commercial centers and large human settlements, and only have access to small trading centers (Fig 1). Key informants were purposively sampled by the study team and had to be working at a wild great ape site and have knowledge of the employee health program at the site. There was no overlap between KIs and participants from BINP. KIs were contacted via email to inquire whether they would be willing to participate between October 31^st^, 2022, and December 28^th^, 2022. Nine KIs were contacted, and four dropped out or declined to participate in KI interviews, while no participants out of the 46 identified by rangers declined to participate at BINP. KIs who did not participate either did not reply to emails or felt they did not have the required knowledge to participate.

#### Phase I: Healthcare access and use for Bwindi Impenetrable National Park staff

Data were collected using a short qualitative individual interview guide (data in S1 Text). Pilot interviews with one Bwindi Community Hospital staff member and two park rangers were carried out before data collection began. Three different groups were included: 1) park wardens, rangers, porters, and security personnel were asked about health facilities available to them, health seeking behavior, and instances when one would take time off work because of illness 2) two senior Bwindi Community Hospital staff members (head doctor or nurse) were interviewed about services provided by BCH, including immunizations, preventative healthcare, and other treatments for conditions prevalent in the area, and 3) a senior member of BINP management was asked about what policies are in place for staff sick leave, how prevention and care services are provided to staff, and what policies are implemented to minimize disease exposure and transmission between staff and gorillas, and what procedures are followed if staff accidentally expose gorillas to infections. Interviews were de-identified by giving participants a number at the time of the interview, and recording their job title, age and gender.

Semi-structured qualitative interviews were conducted among 46 participants in English. Interviews were carried out by the student investigator Maya Homsy King and a research assistant from the Bwindi area. Maya is a White female Master of Public Health Student at the University of California Berkeley, who grew up in Uganda. She has prior experience conducting qualitative interviews in Uganda and has formal training in qualitative analysis. Informed consent and purpose of the study was explained prior to beginning the interview. Interviews were comprised of nine questions and lasted 30 minutes on average. Data were recorded via handwritten notes, and short field notes were made after the interviews each day. No interviews were audio-recorded due to IRB constraints. All interviews took place at participants’ places of work, e.g., ranger stations and visitor centers. Each sector at BINP has a building where rangers report to work, and researchers were given an empty room or secluded outdoor space to conduct interviews with rangers who were not in the field. No people other than the interviewer and participant were present during the interview, no repeat interviews were carried out, and transcripts were not returned to participants. Thirteen participants were interviewed at Buhoma, 15 at Ruhija, 15 at Nkuringo, two senior staff at BCH, and one senior member of BINP management. Participants at BINP were given a new umbrella for as a token of appreciation for their time while participating in the study.

#### Phase II: Key informant interviews regarding employee health programs at other wild great ape sites

Key informant (KI) interviews were conducted with individuals who are knowledgeable about the employee health programs at their great ape site using a qualitative interview guide (data in S2 Text). Individuals were contacted if: 1) they worked at a wild great ape site; and 2) had knowledge of employee health protocols at their place of work. KI’s sites included national parks, a research reserve and a rehabilitation center (Table 3). Key informants were given a list of questions (S2 Text) and the informed consent document for participants to read and sign. Five interviews were conducted, and transcription software was used to transcribe the interview. No repeat interviews were carried out and transcripts were not returned to participants. Interviews lasted 30 to 40 minutes. Questions were centered around employee health practices at participants’ places of work and included information on preventive healthcare practices at wild great ape sites, PPE regulations, and what healthcare is available to employees.

After analysis of the data, a summary of recommendations and findings was submitted to the Chief Warden at BINP, the Research and Monitoring ranger, and head rangers at each participating sector. Unfortunately, it is impossible to ensure that individual participants receive results as we did not retain identifying information for them, but the study team received a detailed response from the Chief Warden acknowledging the results, detailing progress already made on healthcare at BINP, and using the results to find ways forward for all recommendations. The full draft version of this paper has been also shared with the Chief Warden, staff at BCH and all KIs.

### Data analysis

Data were analyzed using qualitative research software Atlas.ti 9.1.3. Thematic analysis was conducted for both BINP and KI components, which is suitable for semi-structured interviews as it enables researchers to structure interview data according to themes and categories, and is useful for understanding the meanings that people give to their experiences and what led them to certain behaviors [18]. Data was first coded according to the question being asked (open coding), then axial coding was used to identify patterns in the data and make connections among codes. Themes were constructed based on participants responses and the employee health guidelines from the IUCN. The results and paper are structured according to the Consolidated Criteria for Reporting Qualitative Research (S1 Table) [19].

Results from interviews at BINP specifically were analyzed thematically based on elements of internationally established employee health guidelines [16]. Themes focus broadly on preventive care (annual physical examinations, infectious disease screenings and deworming), whether and where diagnostic testing is available, the types of facilities used for different conditions, and when staff take time off for sick leave.

## Results

Most respondents were rangers (61%; 28/46) (Table 1). Rangers are employed by the Uganda Wildlife Authority to protect the park, guide tourists into the park and monitor mountain gorillas. Trackers, guides, and security personnel are all rangers, but have slightly different responsibilities and expertise. Most respondents were male (74%; 34/46); the ranger population in Uganda is 80% male. Fourteen porters, who are not directly employed by UWA, were also interviewed. Porters are community members who work part-time carrying bags for tourists who hike into the park.

**Table 1:** Participant demographics from Bwindi Impenetrable National Park. ‘Rangers’ refers to staff who simply described themselves as rangers, and did not mention a specialized role such as tracker, guide, or security personnel.

Occupational category	Participants (n = 46)	Male	Female
Rangers	9	6	3
Trackers	8	6	2
Guides	5	4	1
Security personnel	6	5	1
Porters	14	9	5
Soldier	1	1	0
BINP management	1	1	0
BCH Staff	2	2	0
Total		34	12

### Interviews with Park Staff from BINP

Thematic analysis revealed that employees do not obtain regular physical examinations by a physician, nor do they receive regular deworming. Diagnostics are mostly available at hospitals, not clinics or government health centers and medication is frequently unavailable at health centers. In addition, treatment of chronic or severe illness happens most frequently at hospitals, whereas acute conditions are treated at clinics. Rangers take time off depending on personal opinions of risk to themselves and to gorillas, and assessment of what duties can be performed while ill (Table 2). Each theme is discussed in detail below.

**Table 2:** Themes from interviews with staff at Bwindi Impenetrable National Park.

Theme	Summary finding	Detailed findings
Preventative care	Employees do not obtain regular physical examinations by a physician, nor do they receive regular deworming.	Rangers have insurance that covers services for symptomatic illnesses at selected hospitals, but insurance does not cover physical exams or screenings. Whether employees go to physical exams depends on personal attitudes towards health. Deworming medication is taken only if the individual is experiencing symptoms.
Diagnostics	Diagnostics are mostly available at hospitals, not clinics or government health centers, medication is frequently unavailable at health centers.	Hospitals were most frequently mentioned as providing any kind of diagnostic testing (e.g., for blood, stool, or urine samples). Clinics and health centers were sometimes said to have access to tests for malaria and unspecified sexually transmitted infections. Medication is available at clinics and hospitals, but rarely at government health centers.
Treatment options	Treatment of chronic or severe illness happens at hospitals, whereas acute conditions are treated at clinics.	Clinics are located closer to participants’ workplaces than hospitals and provide faster services than hospitals or government health centers, making them more convenient for acute conditions. Hospitals have more capacity for treatment of severe, chronic conditions and better qualified staff than clinics or health centers. Local herbalists were mentioned as an occasional solution to problems such as cough, flu, malaria. First Aid kits provide free supplies including cough and cold medication for rangers but are frequently understocked.
Sick leave	Rangers take time off depending on personal opinions of risk to themselves and to gorillas, and assessment of what duties can be performed while ill.	Some rangers referred to doctor’s notes and prescriptions as guideline for how much time to take off. Many expressed that it was their own opinion of their condition that guided how much time to take off. Some participants said that if they had mild symptoms, they would do less strenuous work (gate duty, administrative) and rarely said they would go to the forest and interact with gorillas.

#### Employees do not obtain regular physical examinations, nor do they receive regular deworming

All employees of UWA were insured by AAR, an East African health insurance company. Rangers have a comprehensive plan, which most of them say covers all common ailments and treatment for them and their families (up to 4 people), although it did not cover preventive physical exams. Porters, who were classified as ‘support staff,’ were not insured and had to pay out of pocket for services at any private facility. Only select hospitals were enrolled in UWA’s insurance scheme; private clinics were not covered by the insurance scheme. For all rangers other than those stationed at Buhoma (who could walk to Bwindi Community Hospital), private clinics were more convenient; hospitals were over an hour’s drive away. Many participants said they were satisfied with the insurance, and glad that it was available to them, however all participants had to pay out of pocket for any preventive service they requested.

“*They do all the tests for whatever you’re suffering from. If you’re not suffering from anything, and you want to check your condition, insurance doesn’t cover it*.”

Ranger-guide, Ruhija, 39 y/o, F

Therefore, many participants said they only went to a health facility if they had symptoms, and there was no regulation in place mandating physical examinations for any employees. Fewer porters than rangers described going to a health facility for a physical exam. Those who went for physical exams gave reasons such as sexually transmitted infection (STI) testing, especially HIV, and antenatal visits for women.

*“I go there to check myself and some special treatment that are not at the clinics near here*. *I also go to test blood for general issues—if you’re sick and don’t know the cause*. *After 6 or 3 months I would go just to check even if not sick*.*”* Private ranger,

Nkuringo, 32 y/o, M

In addition to the cost, whether participants went for regular physical exams depended on personal attitudes towards their own health or having a chronic condition, such as allergies, that necessitated regular visits to a health facility. Many participants said they only went to a health facility if they were experiencing symptoms, and while a few said they went regularly for physical exams without symptoms.

Finally, porters and rangers said that they rarely, if ever, took deworming medication. Participants said only children took deworming medication at regular intervals. A few said that they took the medication every few months of their own volition, because the health facility reminded them, or when they felt abdominal discomfort. One participant said they used herbs from the forest for deworming purposes.

#### Diagnostics are mostly available at hospitals, not clinics or government health centers, and medications are frequently unavailable at health centers

Interviewees stated that the hospitals they access to consistently had the laboratory facilities and equipment to diagnose a range of conditions, but clinics were more limited. Participants mentioned availability of blood, urine, and stool sample diagnostic testing at hospitals, as well as ultrasound scans, X-rays and a larger number of staff qualified to operate this equipment. Bwindi Community Hospital provided screenings including tuberculosis, blood pressure, and Hepatitis B and STIs for pregnant women, which were covered under the consultation fee. These services were not present at clinics or health centers. At BCH, screening for helminthic infections is done on a symptomatic basis, and triage for respiratory symptoms and temperature happens as soon as patients enter the gate of the facility.

*“If you are pregnant, a Hepatitis B screening is required, as well as other STIs. Some screenings are included in general care- for example, TB screening starts at triage (with a questionnaire), blood pressure is taken.”*

Senior BCH staff, 35 y/o, M

Some clinics had malaria, pregnancy, and STI testing, but some were said to have no testing capacity at all. Clinics were rarely said to have any scanning capabilities. Several participants expressed their wish for better-equipped facilities nearer to their workplaces, especially relating to diagnostic capabilities.

*“If the clinic could test people, they would be doing well. We need scanning, x-rays, fridges for keeping some medicines.”*

Ranger-tracker, Nkuringo, 26 y/o, F

Another important factor in the choice of healthcare facility was the availability of medications. Many people cited lack of available medication at government health centers near them, which is why private clinics or hospitals were the facility of choice for most people. Availability of treatment at clinics was variable; participants at Ruhija frequently described clinics consistently stocking medication, while participants at Nkuringo were more likely to mention that even clinics sometimes had no medication, or only had over-the-counter pain relievers.

*Mostly in clinics you find only one person, or the clinic is closed. We need more clinics; the government could provide more health centers. In most cases, in clinics you can request for tablets, and they don’t have. Mostly they deal with minor illnesses and tablets.*

Ranger, Nkuringo, 37 y/o, F.

#### Treatment of chronic or severe illness happens at hospitals, whereas acute conditions are treated at clinics

**Fig 2:**
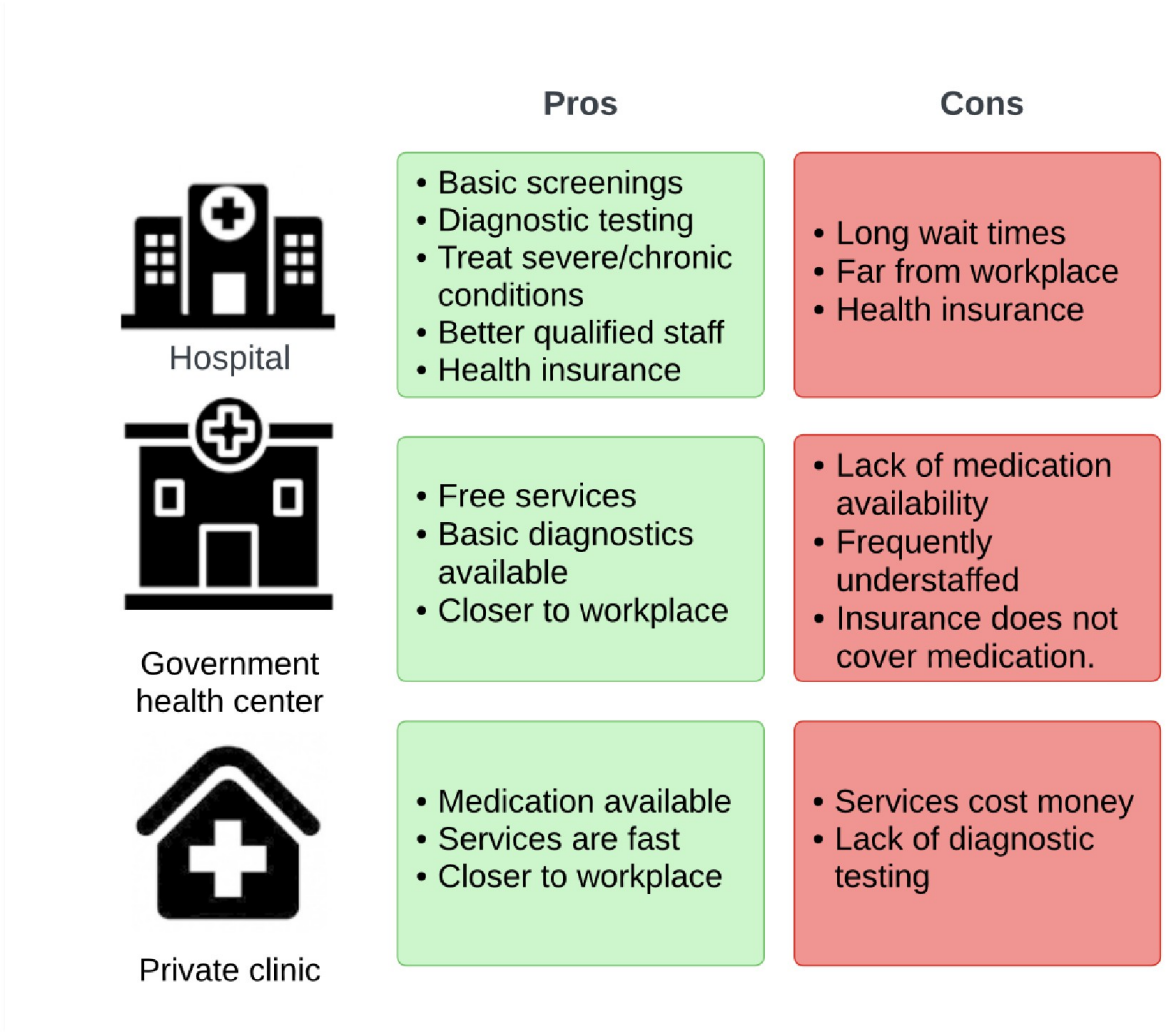
Types of health facilities used by park staff at BINP and their pros and cons according to the majority of participants.

One frequently mentioned recommendation by staff was to have a health provider within the park for BINP staff. Reasons for this included the slow nature of services at many hospitals and the distance staff must travel to reach better equipped facilities, which is especially important when staff need urgent care. The clinic is much faster, and in most cases closer to where the participants reside (Fig 2). The wait time reported at hospitals varied from 30 minutes to a whole day, but participants said that it depended on the time of day, and on how many patients were waiting to receive care.

*“To me, what would be important is that the organization should have clinics within working places. If you fall seriously sick, you have to be rushed to far places. Pregnant staff who are rushed to nearest places sometimes lost the baby. It is hard to find a good facility near the ranger post.”*

Ranger-guide, Ruhija, 39 y/o, F

Participants reported going to the hospital rather than the clinic for issues they perceived to be more serious, except when a condition needed to be treated immediately, in which case they went to the closest clinic (Fig 2). In many cases, despite the fact that it cost money, participants would go to a nearby clinic first, and if a condition was not resolved, they would make the trip to the hospital. Serious conditions were more likely to warrant testing to determine the cause of the illness, and thus was more likely to require a trip to a hospital. The factors of transport availability and cost were mentioned multiple times as limiting factors in accessing better-equipped facilities where insurance is accepted.

*“At clinics, you can go for consultation, history taking, some tests and treatment. At BCH, you can go for tests for different illnesses, HIV, some treatments and medicines. BCH is the most used- you can also access screening for diabetes, hepatitis B, malaria and hypertension.”*

Porter, Buhoma, 43 y/o,F

Participants differed in how they reported reacting to symptoms of malaria; some reported going to the clinic to buy medication, while others would go to the hospital to be tested and treated. All participants perceived ‘general body weakness’, meaning a feeling of fatigue and muscle weakness as serious. Unless it was only ‘mild weakness’, respondents said that body weakness and fatigue warranted staying home from work and seeking diagnosis and treatment.

“[I go to the hospital for] *mostly malaria and fever*. *For cough and flu*, *I visit the clinic*. *Sometimes*, *for small wounds*, *like a cut*, *I go without [going to either the clinic or hospital]*. *When I’m suffering from malaria*, *they get blood*, *take it to the lab and give me drugs*.*”*

Porter, Ruhija, 36 y/o, M

Participants said they usually go to the clinic for severe stomach problems and resolve less severe stomach issues by themselves. Some participants mentioned that they suffered from stomach ulcers, and these sometimes warranted a visit to the hospital. Local herbs were also mentioned as treatments for ulcers in Nkuringo and Ruhija.

*“For ulcers*, *we visit an old woman who can give you a medicine*, *called* ebiculinganyi. *She dries it inside the house and makes it a paste and you drink*. *At the hospital*, *they give you magnesium and if you don’t feel okay after that*, *you can try the herbs*.*”*

Porter, Ruhija, 31 y/o, M

Participants also stated that they sometimes use traditional medicines for the treatment of other ailments, such as cough, flu, malaria, and stomach problems. However, when asked about visiting traditional healers, all participants denied using their services, and mention of traditional healers at times provoked laughter or emphatic denial of the use of their services.

Common ailments including cough, flu, and malaria could be treated using the first-aid kits that UWA keeps at each office in the four sectors of the park. The kit is stocked with medication for common ailments and with bandages. These supplies are free for rangers to use, and many of them mentioned that they would look there first before going to buy medication from the clinic. However, respondents frequently said the kits were not restocked as often as necessary and could be understocked for months on end.

Finally, respondent’s perception of staff and their qualifications, as well as the way they treat patients, was of importance in making decisions for healthcare. Participants said that staff at hospitals were more qualified than those at clinics, which is one reason why more serious illnesses warranted a visit to the hospital (Fig 2). Some participants however mentioned harsh treatment by providers at hospitals because of understaffing.

#### Rangers take time off depending on personal opinions of risk to themselves and to gorillas and assessment of what duties can be performed while ill

Decisions on whether to come to work depended on individual opinions of risk to themselves and to the gorillas, and assessment of what duties could be performed while ill. Some participants mentioned doctor’s opinions or prescriptions determining how long they would stay away from work, and park management described that the length of necessary sick leave was determined by a doctor’s recommendation. Rangers had allowance for up to 6 months of paid sick leave, if necessary, but this must be proven with a doctor’s note. They also have eight days off per month including weekends (regardless of whether they are sick), which are sometimes used to take time off for less severe issues like cough, headache, and gastrointestinal issues. Porters are paid every day they work and not otherwise.

Many rangers said that they would do ‘simple work’ if they had mild symptoms such as headaches or mild cough or flu. Reasons given frequently included the risk of transmission to gorillas, as well as not being able to hike for long periods of time when sick. ‘Simple work’ encompassed office work or gatekeeping, but not going into the forest. Some people said that they would skip work for ‘running stomach’ (diarrhea), as this would make hiking or dealing with clients challenging.

*“Mostly when we are sick, we may even transmit diseases to gorillas, and you may have no energy. When I’m sick, I report to supervisors. Sometimes, they advices me to go to the nearest clinic or hospital. Sometimes they assist with transport if I am in the field.”*

Ranger-tracker, Buhoma, 35 y/o, M

There were some exceptions when people would come to work. A few rangers said that they would go into the forest with respiratory symptoms, including mild cough or cold, but that they would wear a mask.

*“Sometimes I may have headache and still go to work*. *Sometimes I may have cough and flu and still go when it is not strong* [severe]. *If it’s strong*, *I can’t go for hiking*.*”*

Ranger, Nkuringo, 37 y/o, F

Rangers knew that respiratory infections were dangerous for gorillas but mentioned that understaffing put pressure on them to come back to work before they felt fully recovered. It was unclear whether supervisors, a personal sense of obligation, or co-workers were the source of this pressure.

“*It depends on how you feel to come back [to work]. Maybe the pressure at work and the high season can force you to come back*.”

Ranger-guide, Ruhija, 39 y/o, F

### Key Informant interviews: Status of employee health at wild great ape sites in Africa and Asia

KIs were conducted with four sites were in Africa, where gorillas, chimpanzees, and bonobos are endemic, and one site was in Asia, where orangutans are endemic (Table 3). Three out of the five sites included were national parks and overseen by the government. Site 1 had a non-governmental organization (NGO) affiliated with the park that employed their own staff, so employees of the NGO benefitted from NGO healthcare as well as national healthcare. Site 2 was a forest reserve and primarily managed by the research group that started it; it does not have a tourist program, and the only human- primate interaction is for research purposes. Site 3 is a primate rehabilitation center that serves to rehabilitate and reintroduce orangutans to the wild and does not involve tourism.

**Table 3:** Type of site, region, governance of the site, and occupation of each KI is detailed in this table. Site 00 refers to BINP, which was discussed in the previous section of the results.

Site	Type of site	Region	Governance and funding	Approximate number of field staff	Key informant occupation
00	National Park (Bwindi Impenetrable National Park)	East Africa	Government	600	Rangers, porters, park management
01	National Park and affiliated NGO	East Africa	NGO and government	30	NGO-affiliated field veterinarian
02	Forest reserve; research site	East Africa	Government and external funding	15	Director of research site
03	Rehabilitation center	Southeast Asia	NGO	100	Veterinarian, consultant, and advisor for NGOs
04	National Park	Central Africa	Government; external funding for employee health	52	Veterinarian and responsible for health program
05	National Park	West Africa	Government; external funding for employee health	7	Researcher in conservation science

Thematic analysis of occupational health provided at other great ape sites identified four main themes: 1) most sites do not provide for preventive healthcare; 2) staff must travel outside the site to access healthcare; 3) access to diagnostics varies across sites; and 4) all sites have PPE, and clothing requirements for staff, while some have quarantine requirements. Employee healthcare varied greatly per site, depending on funding, location, and type of site (Table 4).

**Table 4:** IUCN employee health guidelines compared to employee health programs at various wild great ape sites.

IUCN Guideline	Site 0 East Africa [Bwindi NP]	Site 1 East Africa [National Park]	Site 2 East Africa [Research Site]	Site 3 Southeast Asia [NGO]	Site 4 Central Africa [National Park]	Site 5 West Africa [National Park]
A periodic, at least annual, physical examination administered by a physician.	Not present	Not present	Not present	Present	Present	Not present
Basic health tests to reveal underlying or chronic conditions that could affect quality of life or life expectancy.	Sometimes present in clinics, present in hospitals farther away	Present at referral hospital 15–30 mins away	Mostly not present. Employees go to clinics on time off, rarely hospitals	Present for HIV and TB yearly	Present with annual physical examination.	Not present
Diagnostic testing (including imaging tests, like chest radiographs) for select infectious diseases, such as malaria, TB, hepatitis and HIV and others of particular concern in the region.	Present at Buhoma, staff must travel far to hospitals from other sectors	COVID tests available, first aid kits available. Lab tests for respiratory syncytial virus (RSV), human metapneumovirus and *S*. *pneumoniae*	Unclear. Site is remote and far from healthcare facilities	Present for malaria, TB, HIV	Present for COVID-19, malaria, hMPV, Ebola, RSV, Mpox and Anthrax	Not present
Verification and/or boosting of immunizations for select communicable diseases of concern for great apes	Not present	Present for meningitis, Hepatitis B, COVID-19	Not present	Not present. Verification and provision only of Hepatitis B vaccine.	Present for Hepatitis B, tetanus, typhoid, and COVID-19.	Not present
Deworming medications administered to personnel and to their immediate families on a quarterly basis, alternating the medication quarterly to reduce the potential for anthelmintic resistance.	Taken as needed from clinics or pharmacies [alternation of medication unclear]	Taken as needed from first aid kit on site [alternation of medication unclear]	Not present	Present [alternation of medication unclear]	Taken as needed [alternation of medication unclear]	Not present
Referral of employees found to have emergency, complicated or chronic conditions to a health care facility or programme for treatment; the minimum Employee health program responsibility should be to ensure that referrals occur and are effective.	Unclear^. All staff have health insurance at select hospitals, but cost of transport is not always covered	Present. Cost of referral to regional hospital is covered	Unclear. All transport for staff is paid for	Present. All staff have health insurance which covers referrals	Unclear. Staff are covered under national health insurance and can access care at the public hospital	Unclear. Treatment is paid for by research group
Health and hygiene education relevant to the location and situation.	Sometimes present. NGOs have provided trainings on prevention of zoonotic disease transmission for some rangers	Sometimes present. Deworming information given to staff, and training on diagnostics for respiratory disease in chimps.	Not present	Present. Information sessions on health and healthcare are given to staff. Normally the topic is on common zoonotic pathogens found in the primate species	Sometimes present	Sometimes present. Guides were trained with material on how to protect great apes from disease
* **PPE and quarantine regulations per site compared to IUCN best practice for disease prevention guidelines**						
Seven days of quarantine after having been sick or traveling internationally	Not present	Not present.	Somewhat present. Quarantine period of 3 days for individuals who leave the site and come back	Not present.	Present. Quarantine period of one week for staff who leave the site and come back	Not present
Change clothes and footwear every time a great ape group is visited, and between great ape groups.	Not present. Staff have a uniform, but it is also used for administrative duties	Present. Unclear about changing between great ape groups	Present. Unclear about changing between great ape groups	Present. Unclear about changing between great ape groups.	Not present. Shoes are cleaned with bleach before and after the forest	Not present
Wear a facemask when coming within 10 meters of great apes.	Present	Present	Present	Present	Present	Present

^ Unclear = information was incomplete

* This section details measures that are not part of the guidelines specific to employee health, but are part of the IUCN best practices for disease prevention for all those interacting with great apes [16].

#### Most sites do not provide for preventive healthcare

Sites 3 and 4 were the only ones that provided staff with an annual health check with a physician. Site 3 described screening for TB and HIV yearly. Site 3 dewormed humans as often as the apes are dewormed, every 3 or 6 months. At Site 4, deworming medication was only given if symptoms were present, similar to all the other sites. Site 4 contracted a doctor from the national health service to provide free annual health checks to park staff including screening for malaria and other infectious diseases such as, TB, COVID-19, respiratory syncytial virus, and human metapneumovirus. The country they are based in also has a national health insurance scheme.

*“Then we collaborate with the doctor, the national doctor from the public hospital. So, we send our trackers and all the people who are working in the park to go in the hospital and the doctor is going to consult them, and to treat them against the disease.”*

—Site 04

Site 4 received funding from a European organization for staff healthcare. Although Site 2 also received external funding, they did not have an annual health check and staff seek services as needed. Site 4 requires park staff, who usually spend two weeks in the field, to take a rapid test for COVID-19 every time they go to the field from camp and one week later.

Three of the six sites (sites 1,3 and 4) mentioned providing vaccinations against Hepatitis B, and COVID-19. Hepatitis B is not currently a concern for zooanthroponotic transmission. KIs from sites 1 and 4 mentioned that provision of the Hepatitis B vaccination was a governmental initiative and happened once people were recruited into the park service. Sites 0, 2 and 5 did not require any vaccinations.

#### Staff must travel outside the site to access healthcare

Staff normally had to travel outside their place of work to access healthcare, except for site 1. Site 1 has a dispensary within the park with employee health workers present. The KI described that this was normally the first point of care for sick employees, and from there they were normally referred to hospitals outside the park.

Site 4 brings in a doctor from the national health system once per year to provide an annual physical exam to employees, but otherwise employees go to the government hospital for their healthcare. Sites 2 and 5 described being especially far from healthcare facilities. Site 2 described being 70 km away from the nearest village or town, meaning employees normally sought healthcare on their time off, as they get two weeks off every six weeks. Site 3 described providing most healthcare through the NGO, although it was not clear where Site 3 was in comparison to the nearest health facility.

#### Access to diagnostics varies across sites

Sites 1 and 4 had access to a laboratory onsite with capacity to test for infectious diseases of concern to great apes, including hMPV and RSV. Other sites relied on access to healthcare facilities outside the park for diagnostic testing. At Site 3 new employees were screened for TB, herpesvirus, HIV, Hepatitis A, B and C, and COVID, and are tested for TB and HIV yearly.

The feasibility of referrals and access to facilities where diagnostic testing and screenings happen depends on the site itself. Some sites, such as Site 1, are closer to urban centers where hospitals and clinics are accessible, whereas others, like Sites 2 and 5 are remote and difficult to access.

#### All sites have PPE, and clothing requirements for staff, some have quarantine requirements

All sites have PPE requirements for staff, with a minimum of a facemask worn when near primates, and with the most conservative requiring that staff change clothes and shoes before and after going to the forest. Site 0 has uniforms for staff, but staff wear these uniforms at the administrative building as well as in the forest. Site 2 has a structure to change clothes on the boundary of the forest, where staff put on forest clothes, and then change into camp clothes after returning from the forest. Sites 1 and 3 also require changing clothes and shoes before going to the forest, or interacting with primates, while Site 4 only implements cleaning shoes with bleach before and after going to the field. Site 5 only requires a face mask, but the primates at this site are not habituated to humans and therefore typically stay far from people.

Site 2 has a quarantine period of three days for individuals who leave the site and come back. During this quarantine period they stay in camp but are not allowed to interact with the chimps. The key informant from Site 2 described that the quarantine used to be five days, but because of loss of staff during the COVID-19 pandemic, they had to reduce it to three days. Site 4 also has a quarantine period of about one week for staff that come back to camp; they admitted it was difficult to implement by did not specify why.

## Discussion

This study aimed to explore the status of healthcare and health-seeking behavior at BINP for park staff working in close proximity to mountain gorillas and learn what employee health programs exist at other great ape sites to provide recommendations for improving healthcare for employees at BINP. Results show that rangers and porters at Bwindi Impenetrable National Park, whose jobs involve daily tracking and visiting of the remaining population of mountain gorillas, are not accessing or achieving the standard of health care that is necessary to minimize the risk of transmission of infectious disease from humans to mountain gorillas and vice versa [16]. Rangers’ health insurance does not cover preventive physical exams, and porters do not have health insurance through UWA. Hospitals that provide diagnostic testing and screenings for risky infectious diseases are far from participants’ places of work, and BINP had no mandatory guidelines on staying home from work for symptomatic staff members.

The primary gaps that could be addressed by BINP are provision of preventive healthcare and health screenings for park staff, and increased access to diagnostic testing. Results from key informant interviews at great ape sites around the world paralleled this finding, revealing that most sites did not expressly provide preventive healthcare to their staff, and only two sites out of six provided an annual health check. Key informant interviews also showed that while employee health programs differ across sites, all sites require use of PPE. The authors’ main recommendations for BINP are to improve preventive healthcare by implementing annual health checks, regular deworming, and access to diagnostic tests for malaria, COVID-19, and other human infectious diseases of potential threat to great apes.

Participants from BINP most often accessed care for acute or less serious conditions at private clinics or government health centers near their workplaces and would make the trip to a hospital for severe or unknown, chronic conditions. This was especially pronounced in Ruhija and Nkuringo, where the two closest hospitals were over an hour’s drive away from rangers’ and porters’ places of work. While insurance covered many of the procedures at these hospitals, staff had to invest considerable time and money to reach the facilities. This was mirrored at many sites in KI interviews. Some sites are very remote and to access a hospital could take hours, meaning that staff normally only go during their time off. Time availability has been found to be a barrier to utilization of appropriate healthcare access in other studies in Sub-Saharan Africa, as it interferes with employment [20, 21]. As a result of this limited access, participants in distant sectors of BINP reported that they seek healthcare for serious conditions primarily at private clinics as a first step, before going to hospitals. Similar findings were seen in Konde-Lule et al.’s 2010 study on healthcare provider choice in rural Uganda [22]. Implications of these include reduced access to diagnostic and testing and preventive care and screening, as these are most often available at hospitals.

Although, screening for health conditions based on demographics and risk profile can reduce the risk of severe disease [23], there was the lack of preventive health services accessible to and sought by rangers and porters, who have strenuous, hazardous occupations that involve frequent interactions with non-human primates in an area of the globe highly susceptible to emerging infectious diseases. Prevention is critical to preventing zooanthroponotic transmission and identifying zoonotic disease before an outbreak occurs. Yet, rangers reported their insurance does not cover preventive screenings and porters have no insurance through UWA. When patients visit a large hospital, like BCH, they are offered free general screenings including TB, blood pressure, and Hepatitis B for pregnant women. However, these exams were not performed in small clinics. Thus, for those who visit hospitals less than annually, it could be years between health screenings. Most sites from KI interviews mirrored this finding. Apart from Sites 4 and 5 that required an annual health check, KIs indicated that staff seek care only when something is wrong. Possible explanations could involve cost, as screenings and physical exams are not always covered by insurance and distance necessary to travel as diagnostic labs and equipment are often available at larger hospitals [24]. Inadequate knowledge or incorrect beliefs about causes and consequences of tuberculosis has also contributed to hesitation to seek diagnosis and treatment in East Africa [24]. Providing regular health education and providing all staff with a free annual physical exam would ameliorate these conditions and improve chances of identifying symptoms and treating them earlier to avoid a zoonotic outbreak among staff or among gorillas.

An important component of preventive care in Sub-Saharan Africa is regular deworming. Most staff at BINP reported only taking deworming medication when they had symptoms of helminthic infection, rather than taking it quarterly, as is recommended by the IUCN [16]. Helminthic infections are of increasing concern in mountain gorillas [25]. Habituated gorilla groups in BINP have been found to have higher helminth egg counts than non-habituated groups, indicating that human interaction may be at fault [26]. Fecal samples collected from the Republic of Congo also showed similar prevalence of multiple helminths including *Necator americanus* and *Enterobius vermicularis* in humans and gorillas inhabiting the same forest [27]. Other recent results show increased helminth egg counts being associated with smaller gorilla group size in Rwanda and the Mgahinga region of Uganda [25]. These findings underscore the importance of providing deworming medication to staff at regular intervals to reduce the prevalence of helminths in the area, thus interrupting potential transmission, and to stop infections in humans at early stages [28].

The situation at BINP supports the finding that unequal access to health insurance and benefits exacerbates the disparity in health outcomes between higher and lower income and educated versus uneducated people in East Africa [29]. At BINP, rangers are salaried and educated compared to porters, who are often subsistence farmers when not serving as porters. Porters were employed only as daily wagers by the park and did not have the same benefits as rangers, even though they were exposed to the same occupational hazards as rangers. Porters in Ruhija and Nkuringo were more likely than rangers to report using clinics for their healthcare than hospitals meaning they are less likely to receive diagnostic testing or screening, as well as be seen by a doctor. On the other hand, porters and rangers stationed in Buhoma had access to the BCH community health insurance scheme that covers several preventive and curative services that other clinics do not. Porters in Ruhija and Nkuringo tended to use other hospitals as they were easier to access than BCH. Health insurance has been shown to improve health outcomes relative to having no health insurance [30], and participants mentioned cost as a barrier to accessing medication and healthcare. KI interviews did not go into detail about whether people with different occupations had differential access to healthcare, except at Site 5, where trackers who were part of a particular indigenous group had free public healthcare while field assistants, who were not part of the group, had to pay for healthcare. However, all staff at Site 5 were provided with a free annual physical exam. At BINP, providing porters and rangers with equal access to insurance would reduce the financial burden on porters and decrease inequalities within the workforce.

Diagnostic testing was not uniformly available across sites, or across sectors at BINP. At BINP, diagnostic testing was said to be more frequently available at hospitals than at private clinics or government health centers. Many participants mentioned clinics lacked malaria or TB tests and have medication for sale without needing diagnostic test results or a prescription. Participants also mentioned that first aid kits at each sector of the park provide injury management materials as well as medication for malaria and coughs free of charge, although they were said to be frequently understocked. This is problematic for several reasons. Symptoms of malaria include high fever, muscle aches, fatigue and flu-like illness [31]. These are relatively non-specific symptoms that could be indicative of any common febrile illness [32]. As such, it is easy to mis-diagnose malaria. While malaria caused by *Plasmodium falciparum* is a major cause of morbidity and mortality in many parts of Uganda [33, 34], prescribing antimalarial drugs without a definitive diagnosis of malaria may result in inappropriate care and poor patient outcomes, as well as increasing antimalarial drug resistance [32, 35]. Assuming that all nonspecific febrile illness is malaria also increases the likelihood that emerging zoonotic diseases will be able to spread in human populations before they are identified. Sites 2 and 5 had diagnostic testing capacity available in the park. Although reagents were limited, they had capacity to test for most common and zoonotically important infections including human metapneumovirus and respiratory syncytial virus. It is critical that employees at wild great ape sites have access to diagnostic testing for symptoms of infectious disease such as febrile illness and respiratory symptoms to improve the accuracy of treatment, minimize inappropriate use of antibiotics, allow for facility-based surveillance, and identify emerging zoonotic disease [36]. A feasible suggestion would be to provide rapid malaria and COVID-19 tests in first aid kits free of charge since these are often the first point of care for staff at BINP.

Finally, although there is ample allowance for sick leave at BINP, many staff reported coming back to work according to their own assessment of what kind of work they could do and how they were feeling rather than using their sick leave to take time off until they had fully recovered. Park management indicated that number of days off should be determined by a doctor’s note or prescription; however, some staff did not mention this when asked how they decided when to start working again. Staff were aware that interacting with gorillas while experiencing a cough or flu could result in zooanthroponotic transmission, but a few staff reported still entering the forest with mild symptoms. Progress has been made by the by the non-governmental organization Conservation Through Public Health (CTPH), who trained 400 staff at BINP in 2020–2021 on measures to prevent disease transmission through masking, hand hygiene and enforcing a 7 meter viewing distance for gorillas [37]. Understaffing was also mentioned as a pressure to come back to work, which may have been one reason for this behavior, however, this may also indicate insufficient education of staff members. To combat this, annual education of staff members, such as the one provided by CTPH, emphasizing the risk of zoonotic and zooanthroponotic transmission should be undertaken.

Limitations of this study include the way in which interviews were carried out at BINP. UWA rangers organized participants based on ranger timetables so as not to disrupt the flow of work. This may have led to selection bias, as head rangers could have recommended participants more likely to give positive feedback about their employer. However, upon review of transcripts, participants provided substantial critical feedback of their employer, including complaints about transport, insurance, and understaffing. This provides evidence that employees were not wary of negative consequences should they criticize their employers. With regard to KI interviews, participants provided information on regulations and facilities at each site, but enforcement of the regulations and use of facilities was not assessed. Additionally, all interviews were carried out in English. Although all participants and KIs were fluent in English, English was not all of their mother tongues, and results may have lost nuance or complexity. Since participants’ work involves daily interaction with English-speaking tourists, they speak English quite well and language did not appear to be a barrier during the interviews. While it is possible that participants would have shared more information had the interview been conducted in their mother tongue, limited study funding precluded interviews in languages other than English. Should participants have needed clarification on words or phrases, a research assistant who spoke Lukiga and Lufumbira was available.

### Recommendations for the employee health program at Bwindi Impenetrable National Park

The results of this study suggest that employees need easier access to preventive care, screenings, and diagnostic testing to ensure timely identification, response and containment of zoonotic and zooanthroponotic disease transmission in humans and gorillas.

Many of the issues raised by staff could be addressed by opening a small clinic or nurse’s station within the park stocked with supplies including injury management and medications and rapid tests for common conditions, such as the practice at Site 1. Several participants mentioned that they would like a health provider to be employed specifically for staff of BINP. If a doctor or nurse were present at each sector, this would improve emergency services, first aid services, and enable staff to receive expert advice and referrals should they be necessary. This clinic should serve porters as well as rangers, so that they can seek care when needed and participate in regular check-ups, reducing the risk for zoonotic and zooanthroponotic disease transmission.

Additionally, all staff, including porters, should be provided with deworming medication at quarterly intervals, and to alternate the type of medication quarterly to mitigate potential resistance in keeping with the guidelines on great ape health [16]. Deworming pills are widely available and cheap in Uganda. Deworming medication should be kept in the first aid kits at each site, and a designated individual, such as a head ranger or healthcare provider, should be responsible for reminding employees at the appropriate intervals.

First aid kits should also be stocked with rapid malaria tests and COVID-19 tests, and if possible a joint COVID-19, flu and RSV assay [38]. This would improve accessibility of diagnostic testing for the common symptoms of malaria and rule out COVID-19 for respiratory illness symptoms. Providing these tests would also reduce overuse of antimalarials and provide more information to staff for how to manage their illness. Both tests are easy to administer to oneself. Malaria tests are widely available, and Uganda recently came out with a COVID-19 rapid test [39]; international tests are also available.

In addition to stocking first aid kits at BINP with deworming medication and rapid tests, training staff on first aid could empower them on how to manage early signs of infectious disease using the materials in the first aid kits, and when to seek further diagnostic testing and treatment. UWA, or equivalent agencies in other countries could contract Bwindi Community Hospital, or nearest large hospitals to provide first aid trainings once a year at each sector in the park for rangers and porters. Finally, health camps, or outreach programs for regular screening of risky infectious diseases, as well as other pertinent conditions would be a feasible way to provide preventive care that does not necessitate long travel, high costs and taking time off work. These outreach programs could also include refresher trainings on zoonotic and zooanthroponotic diseases and safe practices for working in the forest.

To identify the emergence of new zoonotic diseases or zoonotic outbreaks, hospitals and clinics utilized by park staff could keep records of symptoms or conditions of staff and compare them to symptoms and conditions of surrounding community members. This would be useful data in terms of emerging infectious diseases, and common occupational hazards. It was made clear by the BCH staff interviewed that no effort had been made to formally record conditions presented by park staff versus community members, but one member of staff indicated that BINP staff more commonly presented with animal injuries than community members, while the other BCH staff member interviewed had observed no differences. To better tailor occupational health services, larger scale data on what park staff most commonly suffer from would be invaluable.

This study provided an illustration of the One Health factors and linkages at work affecting the health of BINP staff, and employees at wild great ape sites around the world, which in turn has implications for the health of the apes they are aiming to protect. Key recommendations for preventing zoonotic and zooanthroponotic diseases include opening a small clinic or nurse’s station within the park, providing deworming medication and rapid tests in first aid kits and keeping records of park staff’s reported conditions at hospitals.

## Supporting information

S1 Text Interview guide for Bwindi impenetrable National Park Staff. This is the guide used for conducting semi-structured interviews with staff at BNP.
(DOCX)

S2 Text Interview guide for Key Informant interviews. This is the guide used for conducting semi-structured interviews with key informants from great ape sites in five different countries.
(DOCX)

S1 Table Reporting guidelines. This paper used the Consolidated Criteria for Reporting Qualitative Research (COREQ) checklist to ensure all necessary components have been included.
(DOCX)
