# Supplementary material for: Preventing zoonotic and zooanthroponotic disease transmission at wild great ape sites: Recommendations from qualitative research at Bwindi Impenetrable National Park
Source: PLoS One. 2024 Mar 1;19(3):e0299220. doi: 10.1371/journal.pone.0299220 (PMC10906881; doi:10.1371/journal.pone.0299220)
Supplement: S1 Text — This is the guide used for conducting semi-structured interviews with staff at BNP. (DOCX) [file pone.0299220.s001.docx]

# S1 Text: Interview Guide for Bwindi Impenetrable National Park Staff

**Interview guide for employee health survey at BNP**

Part I: Wardens, rangers, porters, security personnel

Occupation, age

1. What are the main health facilities near you?
   1. Traditional healers, clinics, pharmacy?
2. What are the services available at these facilities?
3. Which ones do you use most frequently?
4. About how often do you use these facilities?
   1. More than once a month
   2. About once a month
   3. A few times a year
   4. About once a year
   5. Infrequently (less than once a year)
5. For what conditions do you decide to seek healthcare [at one of the facilities mentioned above]? Why these conditions?
   1. i.e., when you have a cough? Flu? Fever? Something more serious?
   2. Do you use different healthcare modalities for different illnesses?
6. Please describe in detail what happens when you go to the health facility
   1. How long do you have to wait? What happens when you see a healthcare provider?
7. What kind of illnesses or sicknesses would you still go to work with?
   1. For flu, for stomach aches?
8. How do you decide when to come back?
9. What do you think of your access to healthcare?

Part II: Bwindi community hospital

1. What are the most common illnesses people [from the park] come here with?
2. Do you administer vaccinations here? If yes, what kind?
3. If someone comes here with a flu-like illness, what is the standard protocol?
   1. Prescribe medication? Test for malaria?
4. Does anyone receive an annual exam?
   1. i.e. park staff?
5. Do you offer any preventative care? Deworming etc.
   1. Screening for certain conditions every year?
6. How do park staff pay for their care?

Part III: Park management

1. Does UWA have rules for taking time off because of sickness? What are their policies if staff fall sick?
   1. What are the guidelines for when staff who fall sick can come back to work?
   2. Why are the policies this way?
2. Does UWA offer paid sick leave?
   1. Do staff take time off when they are ill?
3. What are the policies in place if a member of staff exposes wildlife to a potentially risky infection?
4. What do you think of staff access to healthcare here at Bwindi?

# 
