# Supplementary material for: Preventing zoonotic and zooanthroponotic disease transmission at wild great ape sites: Recommendations from qualitative research at Bwindi Impenetrable National Park
Source: PLoS One. 2024 Mar 1;19(3):e0299220. doi: 10.1371/journal.pone.0299220 (PMC10906881; doi:10.1371/journal.pone.0299220)
Supplement: S2 Text — This is the guide used for conducting semi-structured interviews with key informants from great ape sites in five different countries. (DOCX) [file pone.0299220.s002.docx]

# S2 Text: Interview Guide for Key Informant Interviews

Occupation:

1. What is the nature of your work with great apes, and can you tell me about the site where you work?
   1. How many people work at the site?
   2. What kind of work do they do?
   3. Who comes into close proximity of wild great apes?
2. What regulations are in place at your place of work/the place you are affiliated with for people who are coming into close proximity of great apes?
3. Is there an employee or occupational health program at the site where you work? If so, please describe how it works.
   1. Are there special requirements for people who are working in close proximity to wild great apes?
   2. Are employees examined by a physician on a regular basis? Do they have access to preventive health measures such as vaccines, regular de-worming, etc.?
   3. What happens if an employee shows up to work complaining of illness? (e.g., a fever or other symptom of a potentially infectious disease?)
4. What kind of preventive health screenings do you recommend in your place of work?
5. What PPE are employees required to wear when they are within close proximity of wild great apes? Under what conditions or for what scenarios is PPE required?
6. [What healthcare measures would you prioritize for an employee health program in a great ape context?] ** ask if in response to #2 there aren’t any precautions required.*
   1. E.g., deworming vs. TB screening, vs. COVID screening and sensitization?
7. In your opinion, what are the greatest disease risks to great apes in the wild right now?
